# The Determinants of Telehealth Provision: Empirical Evidence from OECD Countries

**DOI:** 10.3390/ijerph18168288

**Published:** 2021-08-05

**Authors:** Fuhmei Wang, Jung-Der Wang

**Affiliations:** 1 Department of Economics, College of Social Science, National Cheng Kung University, Tainan 701, Taiwan; 2 Department of Public Health, College of Medicine, National Cheng Kung University, Tainan 701, Taiwan; 3 Department of Occupational and Environmental Medicine, College of Medicine, National Cheng Kung University Hospital, National Cheng Kung University, Tainan 704, Taiwan

**Keywords:** ageing, telehealth, e-health, per capita GDP

## Abstract

Health services provided through the telecommunications system aim to improve the population’s health and well-being. This research aims to explore what digital, economic, and health factors are associated with the provision of telehealth services, especially in ageing communities. Applying Organization for Economic Cooperation and Development (OECD) countries’ experiences, this research tries to construct a logistic regression model between adopting a telehealth system or not, a binary outcome variable, and a group of potentially explanatory variables. Estimation results showed that there were thresholds for telehealth provision: The demand for telehealth service usually began when the provision of telecommunication accessibility reached 50%, the proportion of elders exceeded 10%, or the proportion of health spending occupied more than 3–5% of the gross domestic product (GDP); the slope of each variable seemed to correspond with an increase in demand for such a provision. A growing number of individuals in OECD countries are now readily served by telehealth systems under the COVID-19 pandemic. These findings could be regarded as a model for other countries for implementing the necessary infrastructure early on when any of these parameters reaches its threshold. Moreover, telehealth applied in developing countries could be elevated for wider populations to access basic health services and for the remote delivery of health care. A rational decision could be made to appropriately use additional resources in telehealth provision. With accessible e-health services, the population’s health could be improved, which in turn would possibly increase productivity and social welfare.

## 1. Introduction

### 1.1. Background

The coronavirus disease 2019 (COVID-19) pandemic has inevitably affected the conditions of healthcare provision [1,2]. Many health services have shifted to telecommunication systems to minimize exposure to infectious agents [3,4]. Another advantage of telehealth for the population or patients is in reducing their transportation time and costs [5,6]. These technologies are favorable to populations living and/or working in rural or remote areas, offshore islands, and those who are physically challenged in mobility [3,4,7]. Telehealth aims to act as an efficient tool for developing integrated systems of care [7,8]. By adopting new digital technologies including artificial intelligence [5,6,7,9,10], telehealth facilitates the connection and integration of different health services in a seamless way through the exchange and storage of real-time information across different spectrums of income, age, location, economic resource, and health state [11]. These expected and unexpected changes bring about opportunities and challenges regarding the accessibility of telehealth in times of a pandemic. This research aims to explore what factors would be associated with the provision of telehealth and the results could provide health system administrators, policy makers, and government officials with important determinants for improving health interventions.

### 1.2. Existing Studies

The use of telecommunications technologies in health care is indispensable and transformative from a global perspective. Though this care may be an effective substitute for many conventional in-person healthcare services, the patient’s satisfaction and overall effectiveness deserve more attention and long-term evaluation [9,10,12]. In general, optimal provision of telehealth is associated with good economic performance, quality of life, and social welfare [13,14]. An extensive amount of literature on telehealth assessment has been published. Some is focused on the perceptions of patients and clinicians in medical informatics studies [15,16]; many others concentrate on the efficacy or the financial effects of telehealth in remote healthcare networks [17,18]. While some may discuss the determinants for telehealth implementation, simultaneous comprehensive exploration of such factors seems to be lacking. Moreover, no studies have explored if any thresholds exist for each of these determinants when a country begins to invest in telehealth implementation.

Many factors affect telehealth implementation, such as geographical factors [19,20], health system administrators [21], government officials [22], buy-in by physicians [23], and so on. Researchers have discussed the potential impacts of demographic factors on healthcare funding and urged governments to strengthen conventional in-person and telecommunications healthcare provisions [24,25,26]. Among these, rapidly ageing and aged societies generally affect the way in which health care is organized, delivered, and financed [27,28,29]. As a large proportion of healthcare resources are expended on the elderly, increased telehealth spending could possibly result in a reduction in conventional health expenditure in such societies. With telehealth, older adults do not need to travel so often to engage with health professionals and can do so at home through telecommunications equipment.

Based on the above cited factors, this research uses internet penetration rate to explore the influence of geographical factors on telehealth implementation [7]. Per capita tax revenue or payment represents the potential effects of government sector and administration on telehealth provision [30]. In real-world data, countries with higher incomes often have more demand for health services [31]. This research uses per capita gross domestic products (GDP) to represent the wealth factor. We used the ratio of total health spending to aggregate GDP to explore the possibility of physicians’ buy-in telehealth as a complement to conventional face-to-face health care to improve the population’s health [29,32]. We also used the health indexes of life expectancy, morbidity, and the mortality rate of non-communicable disease to evaluate the influences of health status on the demand for telehealth [33]. Since internet accessibility, income, per capita tax revenue, health indexes, and ageing factors have been identified as key factors for the success of implementing telehealth, the research will first estimate the influences of these predictors on telehealth provision through logistic regression analysis and then depict the relationship between these influential factors and telehealth implementation.

The World Health Organization (WHO) distinguishes telemedicine from telehealth. The former is related to curative medicine and the latter corresponds closely to the field of public health [34]. However, telemedicine can be applied to health promotion, prevention, and public health and should not be limited to curative health care. The distinction could become unnecessary in the long term [35]. Especially, stemming from the rapid penetration of mobile phones, mobile health or m-health has emerged in the recent two decades. We will use the terms telemedicine, telehealth, e-health, and m-health interchangeably in this research.

Recognizing that the field of e-health is rapidly transforming the delivery of health services and systems around the world, the WHO urged Member States to plan for appropriate e-health services in their countries in 2005 [36]. Telehealth services aim to provide accessibility with less time and without geographic barriers. While the wider uptake of digital technologies, including in the healthcare sector, can be hindered by digital divides among different ages, incomes and health statuses [37], these divides reflect barriers in accessing or abilities to adopt telehealth systems in different communities rather than personal preferences among different groups. 

### 1.3. The Provision of Telehealth Services Is Related to Digital and Economic Factors

A growing number of individuals in Organization for Economic Cooperation and Development (OECD) countries are now comfortably using digital technologies in other industries and expect the same level of responsiveness and convenience in health care. Based on OECD countries’ experiences, the present work investigates the influences of the aforementioned factors on the adoption or implementation of telehealth services. This research utilizes rigorous scientific examinations to test: (1) what demographic, fiscal, and health factors affect the provision of telehealth services, (2) how telehealth implementation is associated with these determinants, namely, asking the question ‘Is there a potential threshold value for a country to begin adopting such a system?’ Study results could serve as a guide for the design of telehealth services in ageing societies and public health policies. Our study contributes to and improves on earlier work in several ways. First, we included digital penetration, income, and health status as factors for designing telehealth systems in addition to the ageing population and conventional health services [24]. Second, this study is among the first to use logistic estimation to examine the possibilities of providing telehealth to meet people’s healthcare needs. In fact, logistic models with a binary outcome have been widely applied in studies of epidemiology and health care [38]. No studies have explored if any threshold exists for each of these determinants when a country begins to invest in telehealth implementation. Third, we describe e-health as a tool that enhances interest in public health policies in the community. 

In the following, we describe the process of establishing the logistic model and how we analyzed the estimation results in the telehealth system. We also discuss the implications of this work in policy making and offer conclusions.

## 2. Materials and Methods

### 2.1. Research Method 

The WHO Global Observatory conducted the third global survey on e-health for all 194 WHO Member States to determine whether they had e-health related national policies in place in support of universal health coverage. Results from the 125 responses show that having an e-health strategy was becoming the norm; with well over half of the countries (*n* = 73; 58%) reporting having an e-health strategy in place [39]. The survey presented which year the strategy of e-health is adopted in each individual country. And once adopted, a country would begin to implement many aspects of infrastructure of telehealth progressively, which coexists alongside conventional health care. The Global Observatory for e-health implemented a range of measures to assure quality. The surveys received from participating countries were reviewed for completeness. Before introducing e-health, the dependent variable is defined as 0, which is changed to 1 after the year when the country began to adopt telehealth strategy. This research uses a logistic regression model to establish a relationship between a binary outcome variable, adopting a telehealth system or not, and a group of explanatory variables. The transformation from probability to log odds was a monotonic transformation. Probability ranged from 0 to 1. Natural logarithm odds ranged from negative infinity to positive infinity. The linkage between telehealth provision and determinate factors was estimated as:

(1) $$Logit[P(Tele|X_{ij})]=\beta_{0}+\sum_{j=1}^{k}\beta_{j}X_{ij}+u_{i}$$

(2) $$Tele=\{\begin{array}{ll} 1\;\mathrm{if}\; & Tele^{*}>0\\ 0 & \mathrm{otherwise} \end{array}$$

*X_ij_* represents a country *i’*s digital, economic, and health factors *j*. Tele* was defined as the propensity or ability to adopt a telehealth system. We defined the log-odds ratio $P_{i}/(1-P_{i})$ as a linear function of the explanatory variables. The logarithm was a natural logarithm:

(3) $$\frac{\partial [\log(P_{i}/1-P_{i})]}{\partial X_{ij}}=\beta_{j}$$

The coefficient of *β_j_* was the difference in the log odds of the explanatory variable.

### 2.2. Data Description

Based on the WHO Global Observatory survey, this research applies OECD countries’ experiences and datasets of the dependent and explanatory variables observed from 2000 to 2017. There were 36 OECD countries, including Australia, Austria, Belgium, Canada, Chile, Czech Republic, Denmark, Estonia, Finland, France, Germany, Greece, Hungary, Iceland, Ireland, Israel, Italy, Japan, South Korea, Latvia, Lithuania, Luxembourg, Mexico, Netherlands, New Zealand, Norway, Poland, Portugal, Slovak Republic, Slovenia, Spain, Sweden, Switzerland, Turkey, the United Kingdom and the United States. Among them, 22 OECD countries reported having an e-health strategy in place (*n* = 22, 22/36 = 61%). Data on whether an e-health strategy was in place was collected from the Atlas of E-health Country Profiles of the WHO [39]. In the following, demographic, fiscal, and health explanatory variables were examined. The ratio of health expenditure relative to aggregate GDP, representing the provision of conventional health services. Though the effectiveness of healthcare funding on telehealth has been heatedly debated [40,41], few studies have investigated the substitutes and complementary characteristics of in-person and telecommunications health care on the population’s health [13]. We were also concerned with the influence of conventional healthcare provision on the introduction of telehealth. Health services are regarded as luxury services, because the increase in health spending is bigger than the increase in national income [42,43]. This study uses the factor of income, per capita real GDP (based on US dollars in year 2010) to examine the influence of wealth or economic performance on telehealth demand [44]. Per capita consumption was used to capture the demand for non-health goods in the community and its influence on telehealth implementation [45]. We used life expectancy, morbidity, and the mortality rate of non-communicable disease as health indicators to examine the impact of the population’s health on telehealth demand [46]; and per capita tax payment to represent government resources [47]. Definitions of the elderly and ageing population have been neither straightforward nor universally applicable in previous studies. The WHO categorized the elderly starting at age 65 [48]. This research used the ratio of the elderly, aged 65+, to the total population to represent ageing status. There were 648 observations for each explanatory variable. The ranges of values were relatively large in this study since the estimated samples were more comprehensive. In our sample, Luxembourg had the highest per capita real GDP (US $107,765.8); Latvia had the lowest per capita real GDP (US $12,049.32). In addition, South Korea and the United States, respectively, had the lowest and highest shares of health expenditure to GDP, at 3.99% and 17.21%, respectively. The highest and lowest ratios of the elderly to the population were in Japan with 27.74% and Mexico with 4.98%. The longest and shortest life expectancies at birth were in Japan and Latvia with 84.2 and 69.14 years, respectively. Based on the world telecommunication indicators of the World Bank, the digital penetration rate is defined as the number of individuals using the internet (% of population). The lowest and highest penetration rates were in Turkey and Greece with 3.76% and 98.26%, respectively. The data for this study was collected from OECD statistics, which offers comprehensive information on health status, socio-economic and ageing concerns for carrying out empirical analyses across OECD countries. All statistical analyses were completed using the statistical software STATA (STATA Corp., Houston, TX, USA) version 12.0. Table 1 summarizes the descriptive statistics and shows the means, standard deviations (Std. Dev.), the minimum, and the maximum of the concerned variables and the Appendix A Table A1 presents the correlation matrix of all variables.

## 3. Results

This study provides quantitative estimates of the impact of factors of concern on telehealth provision. Table 2 presents the estimation results. For a one percent increase in the ageing ratio, the expected change in log odds was 1.83. We calculated this through logarithm, for example: odd (ageing ratio = 0.15)/odd (ageing ratio = 0.16) = exp (1.83) = 6.25. A one percent increase in the ageing ratio, lead to about a 6.25 time increase in the odds of introducing a telehealth system. Telehealth strategies embody the aims of public health promotion, prevention, and chronic illness management [7]. The likelihood of introducing telehealth increased with ageing ratios. For a one percent increase in the ratio of health spending relative to the GDP, there was about a 2.49 time increase in the odds of introducing a telehealth system. Telehealth is not a substitute for in-person health care but can be complementary to conventional health services to improve or maintain the population’s health [13]. Income status had a positive influence on introducing telehealth services. Per capita consumption had negative effects on the odds of telehealth implementation. The possibility of adopting telehealth services decreased with increased consumption of non-health goods. For a one percent increase in the penetration rate, there was about a 1.42 time increase in the odds of introducing a telehealth system. Life expectancy, morbidity rates, the mortality rates of non-communicable disease, and per capita tax payment affected e-health strategies insignificantly. The intercept from Table 2 with no explanatory variable was the estimated log odds of adopting telehealth for a society of interest. We transformed the log of the odds back to a probability and found P = 0. The value of rho represents the cluster effects. There was a 98 per cent difference in adopting a telehealth system, with or without implementation, between OECD countries. Sigma_u was the random-intercept standard deviation and sigma_e was the residual standard deviation of fixed effects. The estimation results are valid and without bias.

### Ageing and Economic Effects on the Implementation of Telehealth Systems

We were interested in the influences of the possible explanatory factors on the binary variable of adopting telehealth or not. Using Equation (1), we adopted the following equation:

(4) $$P(Tele|X_{ij})=\frac{1}{1+e^{-(}{}^{\beta_{0}+\sum_{j=1}^{k}\beta_{j}X_{ij})}}$$

**Figure 1:**
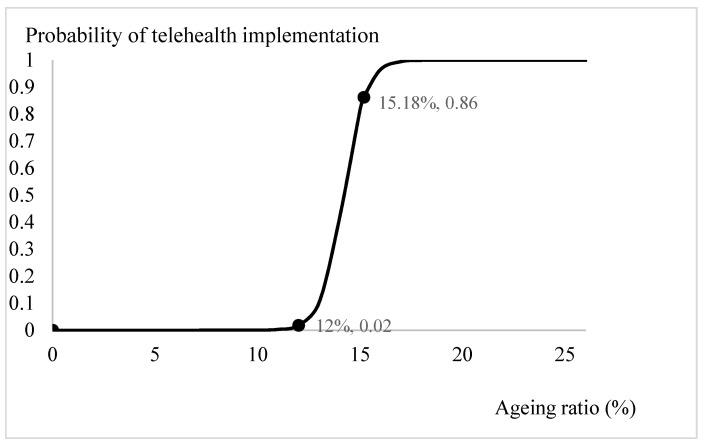
Ageing status and telehealth implementation.

**Figure 2:**
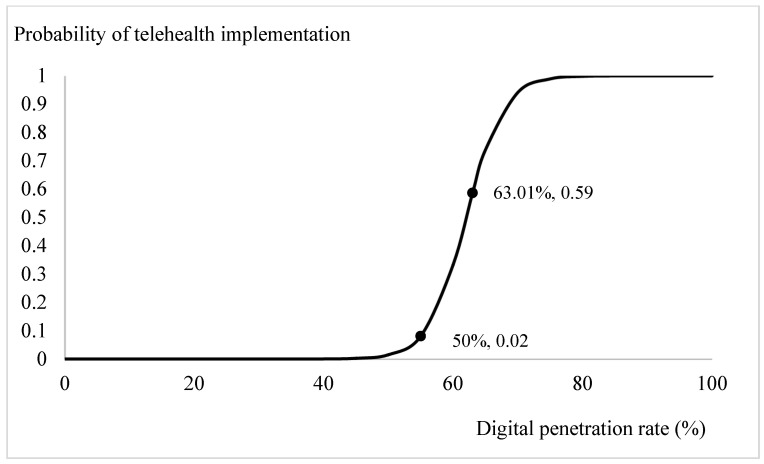
Digital penetration rate (defined as % of population using internet) and telehealth implementation.

**Figure 3:**
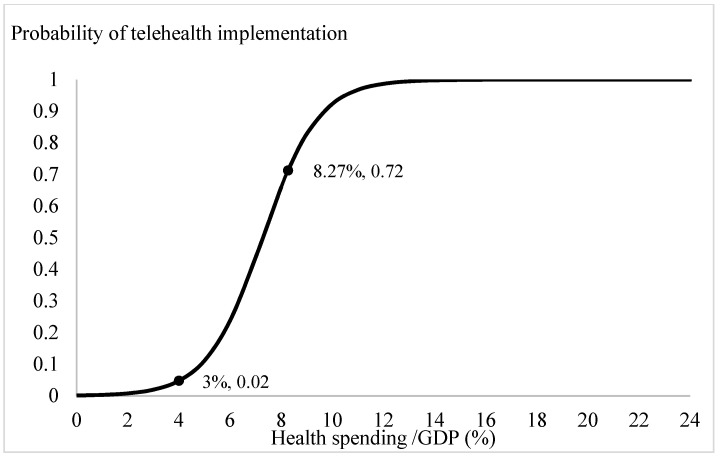
Conventional healthcare provision and telehealth implementation.

**Figure 4:**
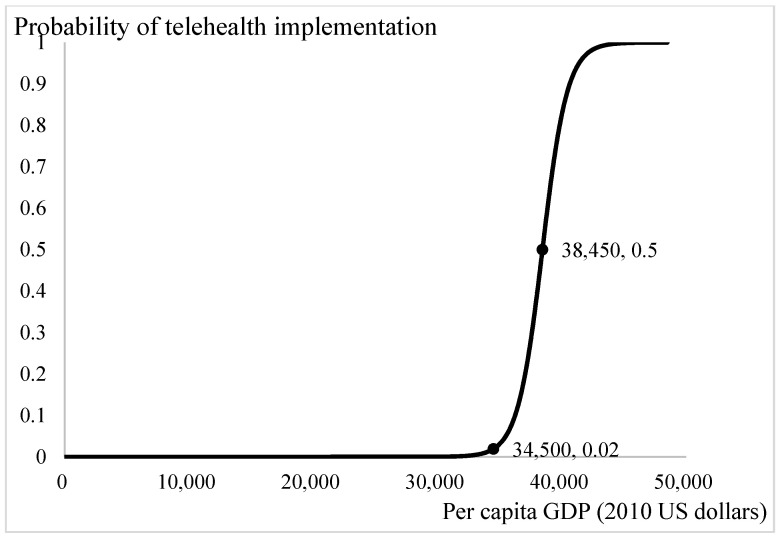
Income and telehealth implementation.

Based on the estimation results from Table 2, we plotted the prospects for telehealth through logit-transformed probability. Apart from the ageing ratio, the influences of the significant explanatory variables, the digital penetration rate, health spending ratio, per capita consumption, and per capita GDP, were specified by incorporating the mean values from Table 1 and the estimated coefficients from Table 2. The trend in the probabilities of adopting telehealth system for different ageing ratios is depicted in Figure 1. When the ratio of the elderly relative to the population exceeded 12 per cent, the probability of introducing telehealth services increased from 0 to 0.02. Especially, when the ageing ratio exceeded 18 per cent, there was a clear demand for telehealth services in the community. With the current OECD countries’ mean ageing ratio, 15.18 per cent, the probability of introducing a telehealth system is 0.86. Through similar calculations, the influences of the variables of interest on the likelihood of adopting telehealth are presented on Figure 2, Figure 3 and Figure 4. Figure 2 shows that the demand for telehealth increases dramatically when the digital penetration rate is greater than 50 per cent and the possibility of telehealth implementation increases from 0 to 0.02. When the penetration rate is above 85 per cent, the community can certainly be provided with telehealth services. With the current penetration rate, 63.01 per cent, the possibility of introducing a telehealth system is 0.59. Figure 3 shows that there is a demand for telehealth when the ratio of conventional in-person health spending to GDP is greater than 3%. Conventional in-person health care should be provided simultaneously to improve the population’s health when the ratio of health expenditure relative to GDP exceeds 13 per cent. With the current health spending ratio, 8.27 per cent, the probability of adopting telehealth services is 0.72. 

Figure 4 presents the influence of income status on the demand for telehealth. The threshold of per capita GDP for demand of telehealth is 34,500 US dollars. When per capita GDP exceeds 45,500 US dollars, there is a definite demand for telehealth among the population. With the current income of 38,450 US dollars per year, the probability of introducing a telehealth system into the community is 0.5. Increases in per capita GDP leads to increases in the affordability of telehealth services. Health services are luxury goods [42]. The poorer the population is, the lesser luxury goods are demanded.

## 4. Discussion and Policy Implications

### 4.1. Countries’ Characteristics Affect Telehealth Implementation

This research examines the proposition that the delivery of health services through telecommunications could be applied across different spectrums of income, age, and health status. Although we found ageing, economic status, and digital accessibility affected the likelihood of introducing a telehealth system into a country, it does not necessary indicate that e-health services are hindered by digital divides among the elderly, across different income statuses, and so on.

We have, however, the following arguments to corroborate the above proposition. First, the factors included in our study were ageing ratio and income, in addition to health status, in-person health care, and digital penetration rates. Because digital technology is based on the infrastructure of telecommunications, the possibility of implementing a telehealth system could be different between countries with various social and economic statuses. After adjusting multiple major determinants in the analysis, we are confident that the logit estimations in this study would be the least confounded. Second, the introduction of telehealth aims to provide accessibility of health care, irrespective of age and rural or urban residents. We understand that telehealth services cannot substitute in-person health care but it would be more suitable for physically challenged patients and/or patients with terminal illness receiving palliative home care [7], who are usually more prevalent in ageing/aged societies. We then examined how the provision of telehealth services is linked to health status and the likelihood of adopting telehealth at different ageing ratios. Third, we plotted the trends in the likelihood of introducing telehealth after adjusting other concerned factors, such as income, in-person health care, ageing ratios and digital penetration rate (Figure 1, Figure 2, Figure 3 and Figure 4). The trends showed in these figures imply that each determinant affects the provision of telehealth according to the experiences of OECD countries and corroborate our proposition. Therefore, we tentatively conclude that telehealth could be designed and implemented early according to the country’s characteristics, and it deserves more attention in national health policymaking.

Based on the experiences of developed countries, mobile communications-based healthcare has improved the living standards of patients [49]. The policies of OECD countries, which advocate universal health care, seems to progressively meet the demands for telehealth, which correspond with the results of Table 2. Because of limited financial budgets and a lack of information and technology infrastructures, telehealth provision in developing countries is usually more expensive and much harder to deploy. However, to meet the demand for universal healthcare coverage including people in remote areas and/or offshore islands, more efforts could be made for similar initiatives in developing countries. A rational decision could be made to appropriately use additional resources on telehealth provision, as opposed to alternative uses such as solving other fiscal problems.

The difficulty that developing countries face may not only be the lack of access to telecommunications systems, but also the lack of well-trained personnel and/or the ability to use technologies. Allowing poorer countries to take advantage of m-health and e-health is helpful to the establishment and sustainability of universal healthcare coverage [39]. Governments could first provide telehealth through emails and pilot projects with lower costs [50,51], and then deploy telehealth as a complement to in-person health care. With accessible and affordable e-health services, the population’s health could be improved, which in turn would be associated with increases in productivity and social welfare.

Although the factors affecting the provision of telehealth might not be exhaustive, our research endeavors to find the major predictors from previous studies and accessible real-world data. For instance, it would be unlikely that including more detailed geographical data on high mountains and/or remote offshore islands, would alter the results of the analysis, as they might already be partially explained by the internet penetration rate. Nonetheless, more studies and investment on telehealth are warranted in the future to continually improve the efficiency and sustainability of the universal healthcare system.

### 4.2. Now Is the Time to Promote Telehealth Implementation

A growing number of individuals in OECD countries are now readily served by telehealth systems to achieve social distancing under the COVID-19 pandemic. Healthcare provided through the telecommunications system is likely to have increased acceptance among policymakers, program developers, patients, and health care providers. Under the COVID-19 pandemic, the elderly, the handicapped, and terminal ill patients at home might live alone, be frail, or be afraid of being infected and delay to access the conventional health services. Providing health care for the disadvantaged groups is one of the properties inherited in the telehealth system. Now is the time to promote such implementation as a tool to give support to healthcare services provided for these groups.

## 5. Conclusions

Based on the estimation results, we have identified the factors that are associated with the implementation of telehealth systems in OECD countries and found the potential threshold value for OECD countries to begin adopting such a system. E-health or m-health can improve health outcome for the majority of rural users also to support the utilization of in-person health care. Establishing telecommunication or m-health infrastructure to provide health services possesses positive externalities for stimulating economic growth by improving health status and enhancing productivity, which in turn leads to better social welfare. Information and communication technologies have contributed substantially to economic growth for developed, developing, and emerging economies [52]. Mobile communications and telecommunications-based health care could be regarded as the key to sustainable economic development and welfare improvement.

On the other hand, in an ageing society, a large proportion of healthcare resources are expended on the elderly. Increased telehealth spending would generally improve communication between healthcare professionals and patients, which would elevate health literacy and the quality of care, especially for those living in remote regions and/or are physically challenged. It is crucial to analyze current policies at the macroeconomic level, which would assist policymakers to properly allocate scarce resources across telehealth and in-person care sectors. Although our results are based on the e-health strategies of OECD countries, the implications of these findings could be applicable to other countries for developing universal e-health care in ageing societies to serve more deserving people. Our research results shed the light on developing countries to implement telehealth early in health care provision when some of these indicators reach the threshold values.

## Figures and Tables

**Table 1:** Summary of Statistics.

Variables	Mean	Std. Dev.	Minimum	Maximum
The elderly/the population (%)	15.18	3.79	4.98	27.74
Life expectancy	78.93	3.10	69.14	84.20
Health spending/GDP (%)	8.27	2.14	3.99	17.21
Per capita GDP (2010 US dollar)	38,450.74	15,898.79	12,049.32	107,765.8
Morbidity (‰)	10.09	5.26	1.7	23.2
Per capita tax payment (2010 US dollar)	14,099.96	6782.02	1012.51	41,874.82
Per capita consumption(2010 US dollar)	19,144.26	9341.55	4181.17	41,636.29
Penetration rate (%)	63.01	23.72	3.76	98.26
Total NCD mortality rate *	425.15	160.54	240.4	3463.6

* Abbreviations: NCD: non-communicable disease (per million persons). Source: Author’s calculations from OECD statistics, http://stats.oecd.org/, accessed on 10 June 2021. WHO: https://www.who.int/data/gho/data/themes/mortality-and-global-health-estimates/ghe-leading-causes-of-death, accessed on 10 June 2021. The World Bank: https://data.worldbank.org/indicator/IT.NET.USER.ZS?end=2015&locations=OE&name_desc=false&start=2000, accessed on 10 June 2021.

**Table 2:** Estimates of multiple logistic regression for telehealth provision.

Study Period = 2000~2017 Number of Observations = 648 Number of Groups = 36 Likelihood-Ratio Test of rho = 0.98: Wald chi2(8) = 184.86 Prob ≧ chibar2 = 0.000				
Telehealth	Coefficient	95% Conf. Interval	Odds	Current Probability
The elderly/the population (%)	1.83 *** (0.43)	0.99 2.68	6.25	0.86
Life expectancy	0.25 (0.29)	−0.79 1.29	-	-
Health spending/GDP (%)	0.91 ** (0.56)	0.14 1.96	2.49	0.72
Per capita GDP (2010 US dollar)	0.001 *** (0.00)	0.0003 0.002	1.00	0.5
Morbidity (‰)	0.16 (0.34)	−0.51 0.83	-	-
Per capita tax payment (2010 US dollar)	−0.0005 (0.00)	−0.002 0.0003	-	-
Per capita consumption (2010 US dollar)	−0.0006 *** (0.00)	−0.001 −0.0001	1.00	0.45
Penetration rate (%)	0.35 *** (0.05)	0.25 0.44	1.42	0.59
Total NCD mortality rate	−0.0001 (0.005)	−0.01 0.01	-	-
constant	−83.17 *** (39.82)	−155.00 −11.32	0	0
/sigma_e	5.10 (0.39)	4.34 5.87		
/sigma_u	12.81 (2.50)	8.74 18.79		
rho	0.98 (0.00)	0.96 0.99		

***: significant at 1%; **: significant at 5%; the numbers in parentheses are standard errors; Abbreviation NCD: non-communicable disease (per million persons).

## Appendix A

**Table A1:** The correlation matrix.

Variables	Telehealth	The Elderly/the Population (%)	Life Expectancy	Per Capita GDP (2010 US Dollar)	Morbidity (‰)	Health Spending/GDP (%)	Per Capita Consumption (2010 US Dollar)	Penetration Rate (%)	Total NCD Mortality Rate	Per Capita Tax Payment (2010 US Dollar)
Telehealth	1									
The elderly/the population (%)	0.15	1								
Life expectancy	0.04	0.19	1							
Per capita GDP (2010 US dollar)	0.26	0.01	0.40	1						
Morbidity (‰)	−0.18	0.01	−0.42	−0.46	1					
Health spending/GDP (%)	0.11	0.23	0.44	0.35	−0.40	1				
Per capita consumption (2010 US dollar)	0.26	0.11	0.54	0.51	−0.52	0.45	1			
Penetration rate (%)	0.51	0.19	0.42	0.47	−0.42	0.28	0.46	1		
Total NCD mortality rate	0.01	−0.08	−0.43	−0.13	0.28	−0.27	−0.29	−0.18	1	
Per capita tax payment (2010 US dollar)	0.13	0.29	0.38	0.52	−0.42	0.29	0.48	0.36	−0.06	1

Abbreviation: NCD: non-communicable disease (per million persons).
